# *Pseudomonas syringae* pv. *tomato* infection of tomato plants is mediated by GABA and l‐Pro chemoperception

**DOI:** 10.1111/mpp.13238

**Published:** 2022-06-10

**Authors:** Saray Santamaría‐Hernando, Álvaro López‐Maroto, Clara Galvez‐Roldán, Martí Munar‐Palmer, Elizabet Monteagudo‐Cascales, José‐Juan Rodríguez‐Herva, Tino Krell, Emilia López‐Solanilla

**Affiliations:** ^1^ Centro de Biotecnología y Genómica de Plantas CBGP Universidad Politécnica de Madrid‐Instituto Nacional de Investigación y Tecnología Agraria y Alimentaria/CSIC, Parque Científico y Tecnológico de la UPM Pozuelo de Alarcón Madrid Spain; ^2^ Departamento de Protección Ambiental Estación Experimental del Zaidín, Consejo Superior de Investigaciones Científicas Granada Spain; ^3^ Departamento de Biotecnología‐Biología Vegetal Escuela Técnica Superior de Ingeniería Agronómica, Alimentaria y de Biosistemas, Universidad Politécnica de Madrid Madrid Spain

**Keywords:** chemoreceptors, entry, GABA, l‐Pro, *Pseudomonas syringae*, virulence

## Abstract

Foliar bacterial pathogens have to penetrate the plant tissue and access the interior of the apoplast in order to initiate the pathogenic phase. The entry process is driven by chemotaxis towards plant‐derived compounds in order to locate plant openings. However, information on plant signals recognized by bacterial chemoreceptors is scarce. Here, we show that the perception of GABA and l‐Pro, two abundant components of the tomato apoplast, through the PsPto‐PscC chemoreceptor drives the entry of *Pseudomonas syringae* pv. *tomato* into the tomato apoplast. The recognition of both compounds by PsPto‐PscC caused chemoattraction to both amino acids and participated in the regulation of GABA catabolism. Mutation of the PsPto‐PscC chemoreceptor caused a reduced chemotactic response towards these compounds which in turn impaired entry and reduced virulence in tomato plants. Interestingly, GABA and l‐Pro levels significantly increase in tomato plants upon pathogen infection and are involved in the regulation of the plant defence response. This is an example illustrating how bacteria respond to plant signals produced during the interaction as cues to access the plant apoplast and to ensure efficient infection.

## Graphical abstract

Perception of GABA and l‐Pro through the PsPto‐PscC chemoreceptor drives the entry of *Pseudomonas syringae* pv. *tomato* into the tomato apoplast.

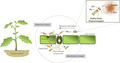

## 1 INTRODUCTION

The phyllosphere is a dynamic habitat where environmental conditions and nutrient availability vary spatially and temporally (Carvalho & Castillo, 2018; Sivakumar et al., 2020; Vorholt, 2012). This imposes a selective environment for bacteria where the ability to cope with adverse environmental conditions and to localize nutrients is a competitive advantage. For many bacterial pathogens, the phyllosphere provides a source of nutrients that are released from the plant interior through leaching and guttation but also through wounds in damaged leaves or via stomata (Ryffel et al., 2016; Vacher et al., 2016). Furthermore, stomata are the main entry points into the plant apoplast for many phytopathogenic bacteria (Melotto et al., 2008; Xin et al., 2018). Once inside the apoplast, the pathogenic stage is initiated. Disease development is thus conditioned by the ability to survive and adapt to the phyllosphere as well as to enter the plant apoplast efficiently. In both processes, chemotaxis plays a key role allowing bacteria to sense and respond to plant and environmental signals (Matilla & Krell, 2018).

Chemotaxis enables bacteria to move in response to chemical gradients (Adler, 1966). In a canonical chemotaxis signalling cascade, perception of a signal by the ligand‐binding domain (LBD) of a chemoreceptor produces a molecular stimulus that alters the autokinase activity of the histidine kinase CheA, which in turn modulates transphosphorylation activity to the response regulator CheY (Matilla & Krell, 2018). When phosphorylated, CheY binds to the flagellar motor, altering its activity. Chemotaxis thus allows bacteria to rapidly adapt to changes in the environment and to actively move towards nutrient sources or signal molecules that inform the bacteria on their environment like hormones or quorum‐sensing molecules (Alexandre et al., 2004; Schulze‐Lefert & Robatzek, 2006). In the context of plant infection, chemotaxis facilitates bacterial access to the apoplast through stomata or wounds and is critical to ensure efficient infection (Matilla & Krell, 2018). In phytopathogenic bacteria, the importance of chemotaxis for efficient plant infection is supported by abundant experimental evidence (Antúnez‐Lamas, Cabrera‐Ordonez, et al., 2009; Antúnez‐Lamas, Cabrera, et al., 2009; Cerna‐Vargas et al., 2019; Hawes & Smith, 1989; Hida et al., 2015; Kumar Verma et al., 2018; Malamud et al., 2011; Matas et al., 2012; Santamaría‐Hernando et al., 2020; Tumewu et al., 2020; Yao & Allen, 2006, 2007). The relevance of chemotaxis is also reflected by the fact that about 90% of phytopathogenic bacteria possess chemosensory genes (Ortega, Zhulin, et al., 2017), whereas such genes are only found in 47% of total bacteria (Sanchis‐López et al., 2021). In addition, phytopathogenic bacteria harbour on average 27 chemoreceptor genes (Lacal et al., 2010), a number that is well above the bacterial average of 13 (Sanchis‐López et al., 2021).

Chemotaxis has been observed in response to root exudates or xylem sap (Verma et al., 2018; Yao & Allen, 2006) and also to abundant compounds in the plant apoplast like amino acids, sugars, organic acids, or phenolic compounds released from wounds (Antúnez‐Lamas, Cabrera, et al., 2009; Ashby et al., 1988; Brewster et al., 2016; Hida et al., 2015, 2017, 2019; Kamoun & Kado, 1990; McKellar et al., 2015; Parke et al., 1987; Tumewu et al., 2020). However, there is scarce information about the signals that govern the switch from the epiphytic to the pathogenic stage in the phyllosphere.

*Pseudomonas syringae* pv. *tomato* DC3000 (PsPto) is a model bacterium to study the molecular mechanisms related to plant infection. This bacterium is the causal agent of bacterial speck in tomato plants and is considered a highly aggressive pathogen once inside the plant (Boureau et al., 2002; Xin & He, 2013). However, it is a weak epiphyte, suggesting that the perception and rapid response of PsPto to plant and environmental signals is critical to ensure entry into the plant apoplast. Stomata are the main entry points of PsPto into the plant apoplast, and previous studies have shown that PsPto is able to move towards open stomata (Melotto et al., 2006). It has also been observed that the mutation of *cheA2*, encoding CheA of the chemotaxis pathway, causes a loss of chemotaxis and reduced fitness on plant hosts (Cerna‐Vargas et al., 2019; Clarke et al., 2016). This bacterium was found to perform chemotaxis towards amino acids, sugars, organic acids, and amides (Cerna‐Vargas et al., 2019; Cuppels, 1988; Kim et al., 2007). Despite the importance of motility and chemotaxis for PsPto phyllosphere colonization and apoplast entry, only one of its 49 chemoreceptors has been characterized. This chemoreceptor, PsPto‐PscA, binds and mediates chemoattraction to d‐Asp, l‐Asp, and l‐Glu and is required for full virulence in tomato plants (Cerna‐Vargas et al., 2019). These results highlight the relevance of amino acid perception in PsPto virulence. Moreover, the saturation of PsPto‐PscA with d‐Asp by its addition to the inoculum reduced virulence significantly. These data suggest that interference with key chemoreceptors is an alternative strategy to fight phytopathogens (Cerna‐Vargas et al., 2019).

Amino acids are implicated in multiple plant processes, including plant defence (Yang et al., 2020), and are abundantly present in the apoplastic tissue, providing a rich environment for the development of phytopathogens (Rico & Preston, 2008). The amino acid composition in the apoplast varies depending on the physiological state of the plant and in response to abiotic and biotic stresses including bacterial infection (Farvardin et al., 2020; O'Leary et al., 2016). This suggests that phytopathogens use amino acids not only as nutrients but also as signals to mediate plant interaction.

After PsPto infection, the concentration of the nonproteinogenic amino acid gamma‐amino‐butyric acid (GABA) increases significantly in *Arabidopsis thaliana* apoplasts (Ward et al., 2010). It has been observed that elevated GABA concentrations down‐regulate the expression of genes that encode the type III secretion system (T3SS), the main virulence factor in PsPto (Park et al., 2010). However, this regulation only occurs once the infection has been initiated (McCraw et al., 2016) and it has been speculated that it may coincide with the increased expression of genes involved in GABA catabolism, as observed in apoplastic populations of *P. syringae* 48 h after infection (Yu et al., 2013). These observations indicate that GABA not only acts as a nutrient for PsPto but also as a signal to regulate its virulence inside the apoplast.

Several chemoreceptors capable of perceiving GABA have been described in different *Pseudomonas* species. The *Pseudomonas putida* McpG chemoreceptor specifically recognizes GABA (Reyes‐Darias, García, et al., 2015), while PctC of *Pseudomonas aeruginosa* perceives preferentially GABA but also binds l‐Pro and l‐His with lower affinity (Rico‐Jiménez et al., 2013). In the phytopathogen *P. syringae* pv. *tabaci* the McpG chemoreceptor is implicated in GABA perception (Tumewu et al., 2020), while in *P. syringae* pv. *actinidiae* (Psa) the PscC chemoreceptor binds GABA and l‐Pro (Ehrhardt et al., 2021; McKellar et al., 2015). The LBD of the PsPto chemoreceptor PSPTO_2448 shares 97.7% sequence identity with the LBD of the PscC chemoreceptor of Psa. Interestingly, in addition to GABA, accumulation of proline has also been observed in *Arabidopsis* plants after PsPto infection (Gupta et al., 2020). It has been reported that this amino acid also plays a key role in plant defence against abiotic and biotic stresses (Verslues & Sharma, 2010).

The PSPTO_2448 chemoreceptor has a dCache_1 LBD domain that contains an amino acid recognition motif conserved in bacteria, archaea, and eukaryotes (Gumerov et al., 2022), suggesting that the perception of amino acids is an important feature throughout the Tree of Life. Considering its conservation and the important role of amino acids as signals for both plants and PsPto, we investigated signal recognition by PSPTO_2448 and its role during plant interaction.

## 2 RESULTS

### 2.1 PsPto‐PscC binds GABA and l‐Pro

**FIGURE 1:**
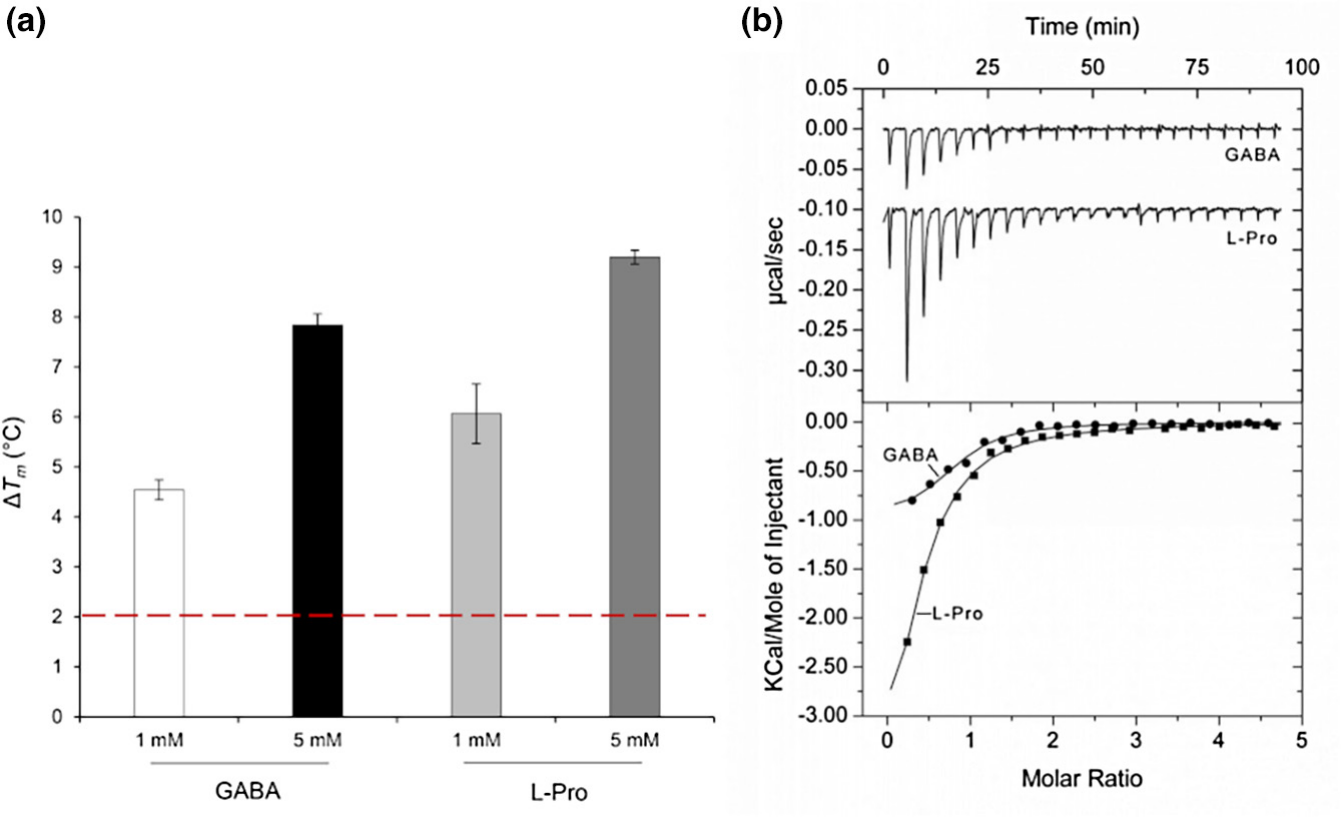
The PsPto‐PscC chemoreceptor binds specifically to GABA and l‐Pro. (a) Differential scanning fluorimetry‐based ligand screening of PsPto‐PscC‐LBD. Shown are the changes in the midpoint of the protein unfolding transition (*T*_*m*_) caused by the addition of 1 mM and 5 mM GABA or l‐Pro with respect to the *T*_*m*_ of the ligand‐free protein. The red dashed line highlights the threshold of 2°C, indicative of binding. Data are the means and standard deviations from three assays. (b) Microcalorimetric studies showing the binding of GABA and l‐Pro to PsPto‐PscC‐LBD. Upper panel: Titration raw data for the injection of 4.2–8.6 μl of 500 μM ligand solutions into 13 μM PsPto‐PscC‐LBD. Lower panel: Integrated, dilution heat‐corrected, and concentration‐normalized peak areas fitted with the one‐binding‐site model of ORIGIN.

To identify the ligands recognized by PsPto‐PscC, its LBD was purified using affinity chromatography and submitted to thermal shift assays. This method records changes in the thermal stability of proteins caused by ligand binding (Martin‐Mora et al., 2018). Increases in the midpoint of the protein unfolding transition (*T*_*m*_) greater than 2°C are considered significant and indicative of binding. The *T*_*m*_ of the ligand‐free recombinant protein was 35.1°C, and significant *T*_*m*_ increases were observed for both GABA and l‐Pro (Figure 1a). In contrast, no significant *T*_*m*_ increases were observed for any of the compounds present in the PM3B phenotype compound array that contain amino acids and many other nitrogen sources (Figure S1). Isothermal titration calorimetry assays confirmed these results as the titration of PsPto‐PscC LBD with GABA and l‐Pro revealed binding with affinities of 1.87 ± 0.3 μM and 4.52 ± 0.4 μM, respectively (Figure 1b). Considering that the *P. aeruginosa* PctC chemoreceptor binds l‐His in addition to GABA and l‐Pro, we also performed microcalorimetric titrations with l‐His. However, no binding heats were detected (data not shown). These results indicate that PsPto‐PscC LBD binds specifically GABA and l‐Pro, as observed for the Psa homologue PscC (McKellar et al., 2015).

### 2.2 PsPto‐PscC mediates chemotaxis to GABA and l‐Pro in PsPto

**FIGURE 2:**
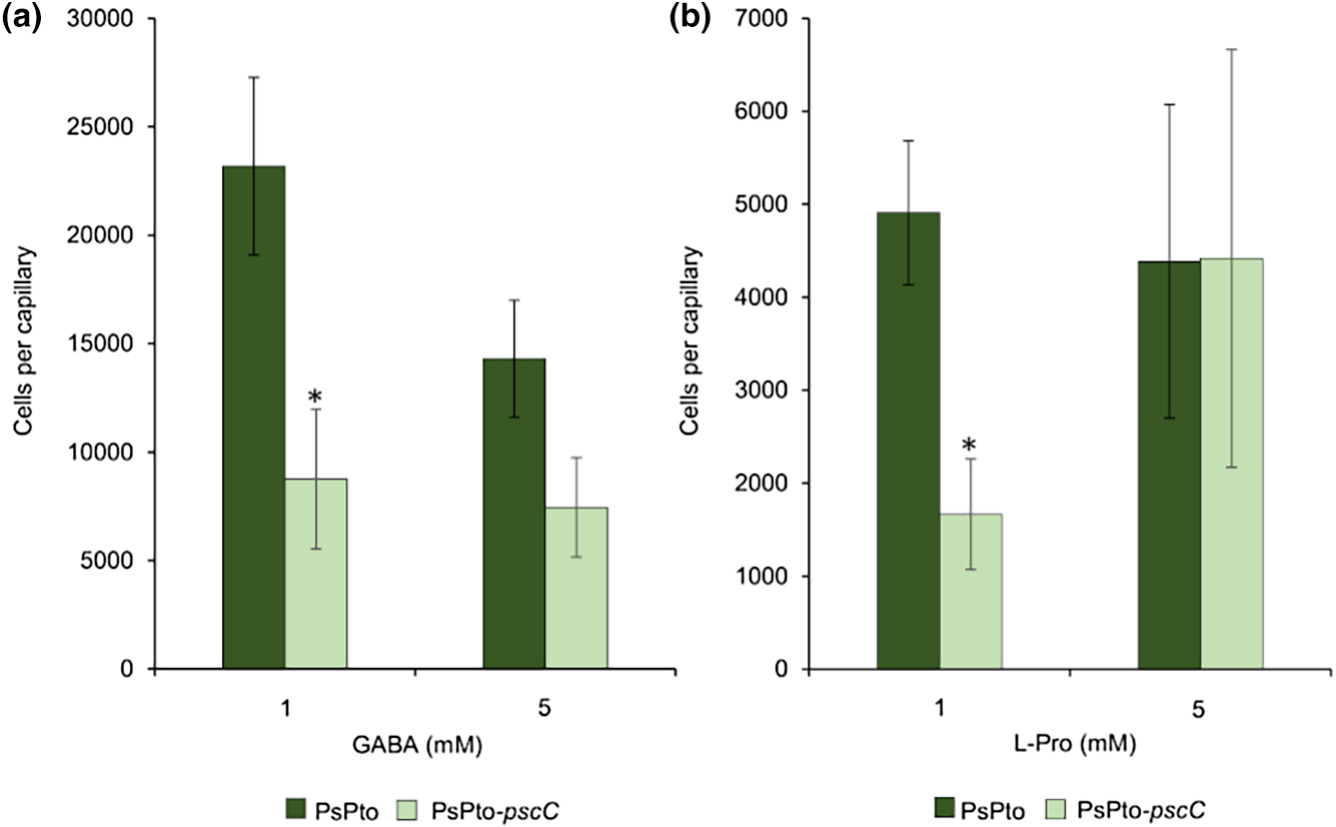
Quantitative capillary chemotaxis assays of PsPto wild type and the PsPto‐*pscC* mutant strains towards GABA (a) and l‐Pro (b). The data have been corrected with the number of cells that swam into buffer‐containing capillaries. Shown are means and standard errors from three independent experiments conducted in triplicate. Values that are significantly different are indicated by asterisks (*p < 0.05).

To evaluate the chemotactic response of PsPto to GABA and l‐Pro, we performed quantitative capillary chemotaxis assays with the wild‐type (WT) strain. The results showed significant responses of PsPto towards these compounds with a maximum chemotactic response at 1 mM (Figure 2). To evaluate whether the chemotactic responses towards these amino acids were mediated by the PsPto‐PscC chemoreceptor, we generated a PsPto‐*pscC* mutant strain. We observed reduced chemotaxis of the mutant strain to both ligands at 1 mM, whereas at a ligand concentration of 5 mM a reduction was only observed for GABA (Figure 2). The observation that there was no complete loss of chemotaxis to both ligands in the PsPto‐*pscC* mutant indicates the existence of other chemoreceptors with overlapping ligand specificities. To verify whether the ligand specificity determined by protein binding studies also corresponds to a chemotactic specificity, we conducted quantitative capillary chemotaxis assays using other amino acids such as l‐Asp, l‐Glu, and l‐Asn. We did not find any significant differences in the chemotactic responses towards these compounds between the PsPto‐*pscC* mutant and the WT strain (Figure S2). Complementation of the PsPto‐*pscC* mutant with *pscC* provided in trans resulted in a chemotaxis phenotype that was superior in its magnitude to the WT strain (Figure S3). Expression in trans from multicopy plasmids enhances cellular chemoreceptor levels and previous studies following a similar approach have also reported an increase in the magnitude of the chemotactic response (Fernández et al., 2016; Hida et al., 2020; Rico‐Jiménez et al., 2022).

### 2.3 GABA perception by the PsPto‐PscC chemoreceptor regulates the expression of genes involved in GABA catabolism

**FIGURE 3:**
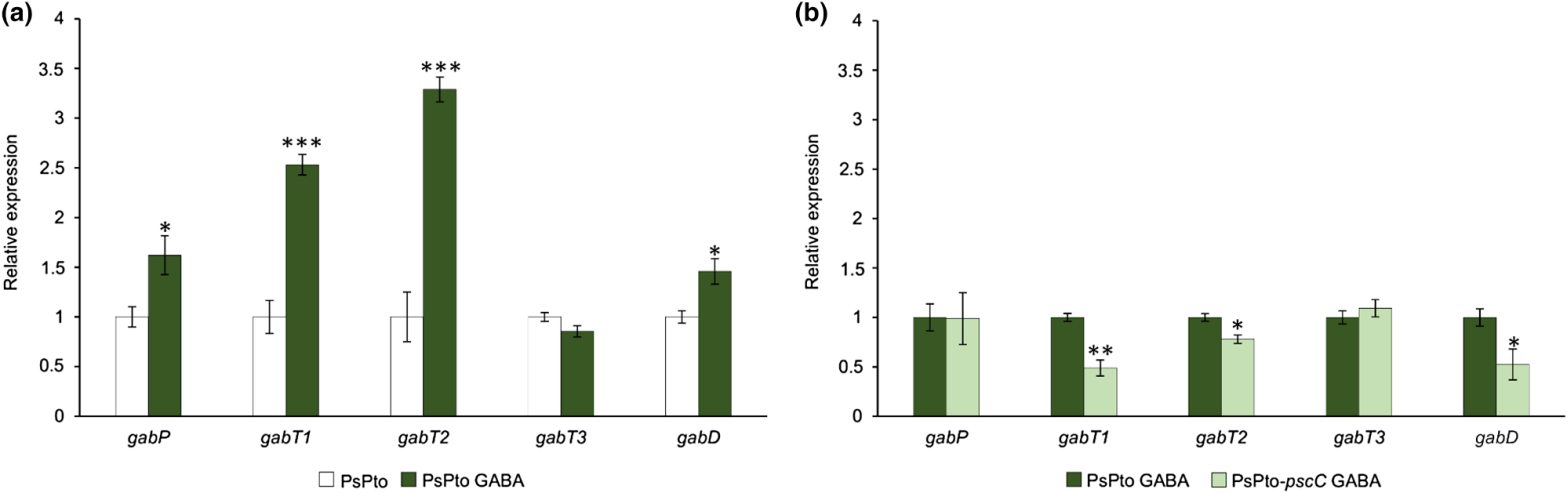
Expression of GABA catabolic genes is regulated by GABA perception through the PsPto‐PscC chemoreceptor. (a) Bars represent the relative expression changes, quantified by reverse transcription‐quantitative PCR (RT‐qPCR), in the wild‐type strain PsPto in the presence (dark green) or absence (white) of 1 mM GABA. (b) Bars represent the relative expression changes, quantified by RT‐qPCR, in the presence of 1 mM GABA in the PsPto‐*pscC* mutant (light green) with respect to the wild‐type strain (dark green). Data represent the means and standard errors of three biological replicates. Values that are significantly different are indicated by asterisks (*p < 0.05; **p < 0.01; ***p < 0.005).

In *P. syringae* pv. *phaseolicola* NPS3121 and *P. syringae* pv. *syringae* B728a the expression of genes involved in GABA catabolism is up‐regulated in the apoplast (Hernández‐Morales et al., 2009; Yu et al., 2013). Considering that GABA is the most abundant amino acid in the tomato apoplast (Rico & Preston, 2008), we assessed whether the expression of genes involved in GABA catabolism was altered in PsPto following the addition of GABA to cells growing in *hrp*‐inducing minimal medium. For these experiments we used GABA at 1 mM, a value close to its concentration in the tomato apoplast of 575 μM (Rico & Preston, 2008). We observed that the expression of the gene encoding the permease GabP was up‐regulated as compared to nontreated WT cells (Figure 3a). GabP has been identified as the principal GABA transporter in PsPto (McCraw et al., 2016). In addition, we evaluated the expression of the three genes encoding GABA transaminases, *gabT1*, *gabT2*, and *gabT3*, and the succinic semialdehyde dehydrogenase gene *gabD*. Our results showed that the expression of *gabT1*, *gabT2*, and *gabD* was up‐regulated after GABA addition compared to nontreated cells (Figure 3a). However, no differences in *gabT3* expression were observed (Figure 3a). These results show that the presence of GABA, in concentrations similar to those present in the tomato apoplast, alters the expression of GABA metabolic genes in PsPto. To determine whether this regulation is mediated by GABA perception through the PsPto‐PscC chemoreceptor, we analysed the expression of these genes in the PsPto‐*pscC* mutant strain. Whereas the gene expression of *gabT1*, *gabT2*, and *gabD* was down‐regulated in the PsPto‐*pscC* mutant strain as compared to the WT strain in the presence of GABA (Figure 3b), no differences in the expression of the *gabT3* and *gabP* genes were observed between the WT and the mutant strain (Figure 3b).

### 2.4 Expression of *hrpL* and *avrPto* is not altered in the PsPto‐*pscC* mutant

**FIGURE 4:**
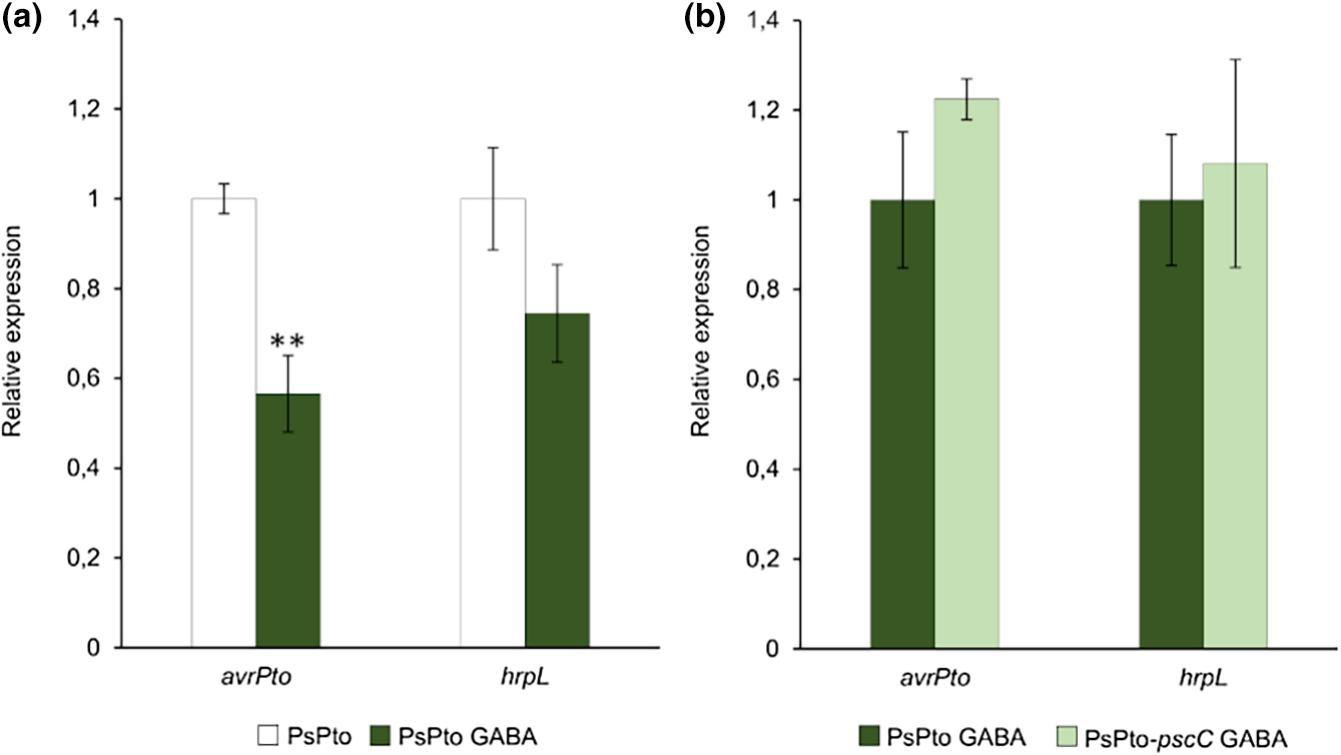
The GABA‐mediated down‐regulation of the type III secretion system‐related genes is independent of PsPto‐PscC. (a) Bars represent the relative expression changes, quantified by reverse transcription‐quantitative PCR, in the wild‐type strain PsPto in the presence (dark green) or absence (white) of 1 mM GABA. (b) Bars represent the relative expression changes in the presence of 1 mM GABA in the PsPto‐*pscC* mutant (light green) with respect to the wild‐type strain (dark green). Data represent the means and standard errors of three biological replicates. Values that are significantly different are indicated by asterisks ( **p < 0.01).

GABA levels increase in the apoplast under biotic stress conditions (Farvardin et al., 2020; O'Leary et al., 2016; Park et al., 2010). This increment has been associated with a greater plant resistance against PsPto infection (Deng et al., 2020; Park et al., 2010). Moreover, it has been reported that increased GABA levels are associated with a down‐regulation in the expression of T3SS‐related genes (McCraw et al., 2016; Park et al., 2010). Considering these previous results, we investigated whether the addition of 1 mM GABA causes a decrease in the expression of the *hrpL* and *avrPto* genes. In accordance with previous studies (McCraw et al., 2016), we observed a down‐regulation in the expression of both genes as compared to nontreated WT cells (Figure 4a). In order to determine whether the regulation of the expression of the T3SS‐related genes was mediated by GABA perception through the PsPto‐PscC chemoreceptor, we analysed the expression of the genes in the mutant and WT strains but did not observe any significant difference (Figure 4b). The data thus indicate that PsPto‐PscC mediates GABA chemoattraction, which in turn may facilitate access to the plant apoplast. However, PsPto‐PscC does not seem to play a role in the regulation of T3SS‐related genes that usually occurs during later stages of the infection.

### 2.5 PsPto‐PscC function is required for full virulence of PsPto in tomato plants

**FIGURE 5:**
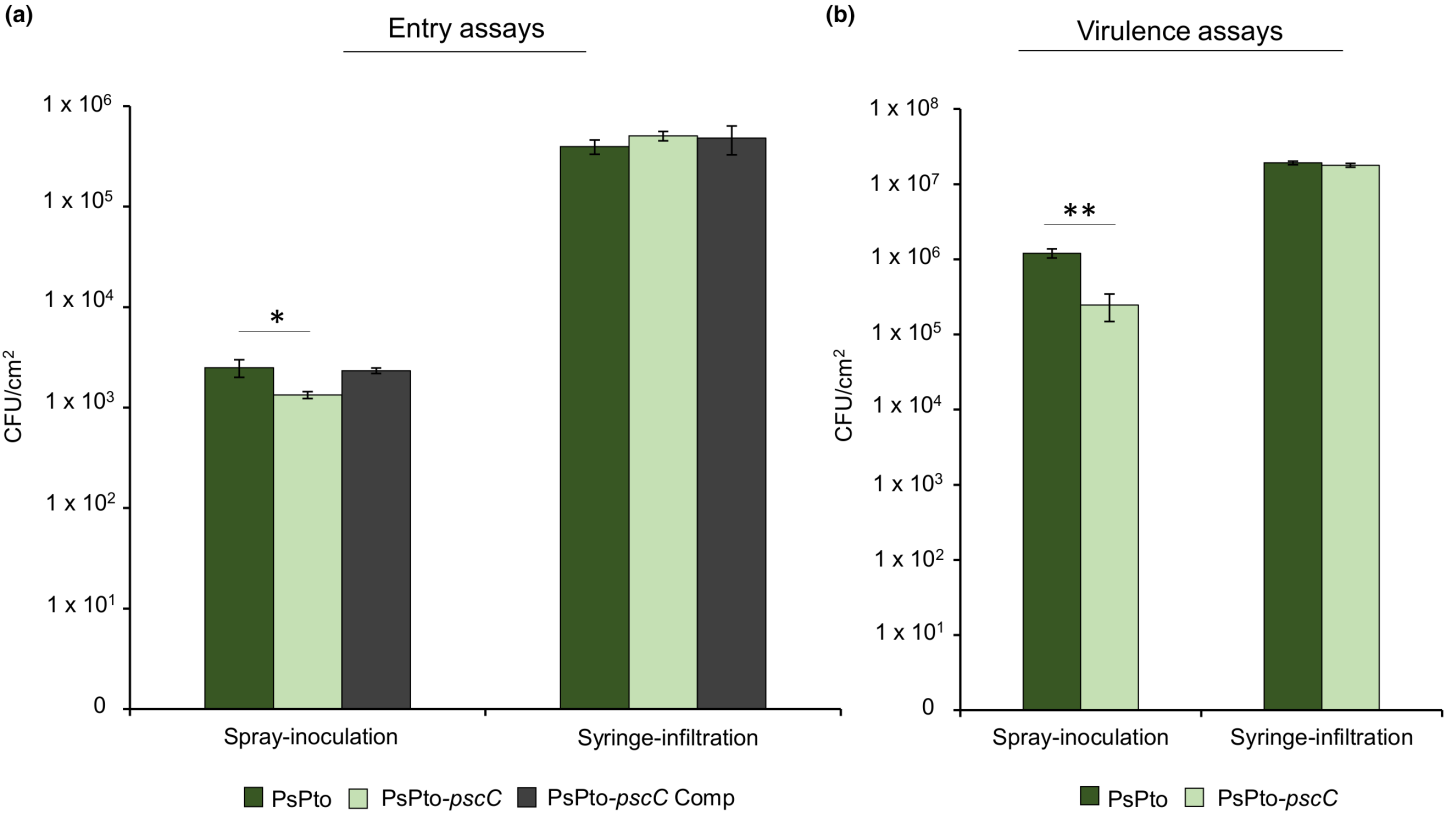
The PsPto‐*pscC* mutant shows reduced entry and virulence in tomato plants. (a) Entry of *Pseudomonas syringae* pv. *tomato* wild type (PsPto), PsPto‐*pscC* mutant, and the complemented mutant (PsPto‐*pscC* Comp) (10^8^ cfu/ml) at 2 h postinoculation after spray‐inoculation or syringe‐infiltration. (b) Bacterial populations of the wild type and the PsPto‐*pscC* mutant at 6 days postinoculation (dpi) after spray‐inoculation (10^8^ cfu/ml) or at 3 dpi after syringe‐infiltration (3 × 10^4^ cfu/ml). Shown are means and standard errors from three independent biological replicates. In each experiment, three plants were analysed by sampling five 1‐cm‐diameter leaf discs per plant. Values that are significantly different are indicated by asterisks (*p < 0.05; **p < 0.01).

Chemotaxis allows bacteria to localize and move towards entry points such as stomata (Alexandre et al., 2004; Schulze‐Lefert & Robatzek, 2006). Considering the reduced chemotaxis of the PsPt*o‐pscC* mutant strain to GABA and the observed alteration in gene expression, we evaluated whether these phenotypes had an effect on virulence in tomato plants. To this end, we conducted both entry and virulence assays to ascertain the role of this chemoreceptor during the initial or later stages of infection. Moreover, we used different inoculation methods, namely the spray‐inoculation approach, which mimics natural entry conditions that bacteria face in the phyllosphere, and the infiltration method, in which the pathogen is placed inside the plant. There is a significant body of evidence demonstrating that chemotaxis‐deficient strains show reduced virulence in spray‐inoculation assays, which is due to that fact that directed motility is required to localize and move towards plant entry points (Yao & Allen, 2006). In a number of cases, analysis of the same chemotaxis mutant strains using infiltration assays, where the pathogen is directly introduced into the plant, did not reveal differences in virulence from the WT (Yao & Allen, 2006). Spray‐inoculation resulted in a significant reduction in the number of PsPto*‐pscC* mutant cells that entered the plant as compared to the WT cells at 2 h postinoculation (hpi) (Figure 5a). This finding is supported by a reduced cell count in virulence assays as measured 6 days postinoculation (dpi) (Figure 5b). However, no significant differences between both strains were observed when plants were infiltrated (Figure 5b). The reduced entry and virulence of the PsPto‐*pscC* mutant strain were restored to WT levels by complementation with the *pscC* gene provided in trans (Figures 5a and S4). These findings thus demonstrate that PsPto‐PscC function is important to drive bacterial entry into the plant apoplast and that its mutation significantly alters infection severity in tomato plants.

**FIGURE 6:**
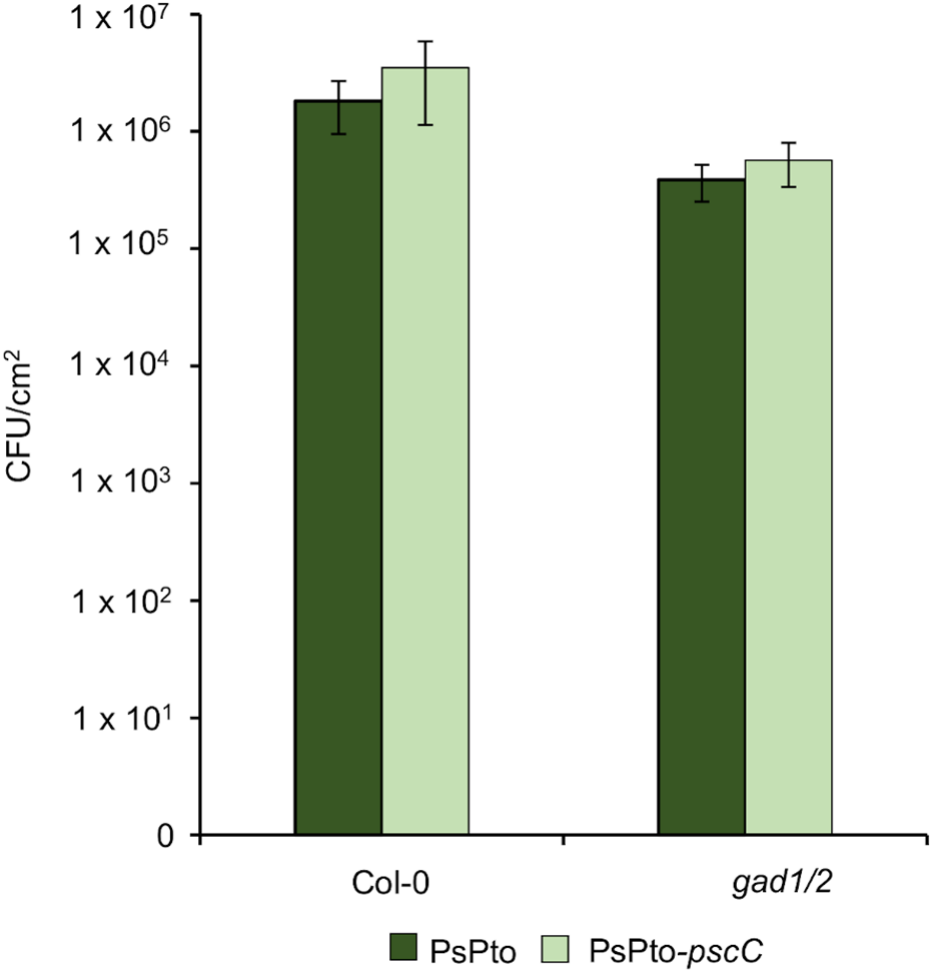
Chemoperception of GABA is not required for *Arabidopsis* PsPto infection. Bacterial populations of wild‐type (PsPto) and PsPto‐*pscC* mutant strains at 6 days after spray‐inoculation (3 × 10^8^ cfu/ml) of *Arabidopsis thaliana* Col‐0 or *gad1/2* plants. Shown are means and standard errors from three independent biological replicates. In each experiment, six inoculated plants were analysed by sampling three leaves per plant.

In order to get further insight into the role of GABA chemoperception during the infection process, we assessed virulence in *A. thaliana gad1*/*2* mutant plants, which present reduced GABA levels (Scholz et al., 2015). Wild‐type Col‐0 and *gad1/2 A. thaliana* plants were spray‐inoculated with the WT and PsPto‐*pscC* mutant strains. Data did not reveal any difference in bacterial populations between the WT and PsPto‐*pscC* mutant strains, independently of the *Arabidopsis* genotype assayed (Figure 6). Moreover, as previously reported (Xian et al., 2020), no differences were observed in infection severity between Col‐0 and *gad1/2 A. thaliana* plants (Figure 6).

## 3 DISCUSSION

Apoplastic fluid has traditionally been considered as an important source of nutrients for bacterial pathogens, allowing their growth and development. Moreover, bacteria have evolved adaptations to exploit changes in plant metabolism induced after microbe perception (Leonard et al., 2017), and, therefore, use apoplastic compounds as cues to regulate the interaction with the plant. Apoplastic compounds can also be considered as signals driving the access of bacterial pathogens to the plant apoplast (Antúnez‐Lamas, Cabrera, et al., 2009; Melotto et al., 2006) and chemotaxis allows the motility‐based entrance of bacteria (Matilla & Krell, 2018).

Multiple lines of evidence indicate that the capacity to sense and respond to amino acids is of crucial importance for many forms of life. (1) A recent study has identified an amino acid‐specific sensor domain, dCache_1AA, that is found throughout the Tree of Life in archaea, bacteria, and different eukaryotes (Gumerov et al., 2022). In bacteria, this domain is found in all major families of transmembrane receptors, including chemoreceptors, sensor kinases, guanylate/adenylate cyclases, cyclic‐di‐GMP phosphodiesterases or serine/threonine kinases and phosphatases. (2) In bacteria, multiple mechanisms have evolved to sense amino acids. Next to a direct recognition at different types of LBDs, namely dCache_1AA, TarH, or DAHL (Matilla, Velando, et al., 2021), there is evidence for transmembrane receptor stimulation by the binding of amino acid‐loaded solute‐binding proteins (Matilla, Ortega, & Krell, 2021). (3) The ligand profiles of many bacterial chemoreceptors have so far been determined (Matilla, Velando, et al., 2021). However, the by far most abundant family is formed by amino acid‐responsive chemoreceptors that show a wide phylogenetic spread (Matilla, Velando, et al., 2021). In the framework of the general importance of amino acid sensing in life, our study illustrates the central role of sensing these ligands in the context of plant infection. In the context of phytopathogens, the amino acid composition of the apoplast and root exudates were found to change significantly after bacterial infection (O'Leary et al., 2016; Yuan et al., 2018). Taking this data together, amino acids may be key players in the signalling process between plants and bacteria during infection.

Only a limited number of amino acid‐specific chemoreceptors have been functionally characterized in phytopathogenic bacteria (Cerna‐Vargas et al., 2019; McKellar et al., 2015; Rico‐Jiménez et al., 2013; Tumewu et al., 2020, 2021). In this work we have identified the ligands of the PsPto‐PscC chemoreceptor of PsPto. We found that PsPto‐PscC binds specifically to GABA and l‐Pro, similar to what was observed for the PscC homologue in Psa (McKellar et al., 2015). However, the characterization of the role of Psa‐PscC during the interaction with the plant has not been addressed in Psa.

In plants, GABA and proline are implicated in the control of carbon/nitrogen metabolism and in the regulation of different aspects of plant physiology (Tarkowski et al., 2020). However, these amino acids also play a key role as signalling molecules during the plant response to different stresses, including pathogen infection (Seifikalhor et al., 2019; Tarkowski et al., 2020). Under stress conditions, GABA levels increase in the apoplast due to the activation of the glutamate decarboxylase enzyme GAD through Ca^2+^ signalling (Tarkowski et al., 2020; Ward et al., 2010). Similarly, l‐Pro levels increase by the activation of the enzyme pyrroline‐5‐carboxylate synthase (P5CS), involved in l‐Pro synthesis from l‐Glu (Mattioli et al., 2009). Moreover, proline can be converted into GABA through a nonenzymatic reaction under stress conditions (Signorelli et al., 2015) and in the case of *Arabidopsis* plants, some GABA transporters are also able to transport proline (Breitkreuz et al., 1999). PsPto‐PscC LBD bound GABA and l‐Pro with high affinity as evidenced by *K*_D_ values in the lower micromolar range, which are comparable to those obtained for PscC from Psa (Ehrhardt et al., 2021) and PctC from *P. aeruginosa* (Rico‐Jiménez et al., 2013). For PctA and PctB, two other amino acid‐responsive dCache‐containing chemoreceptors in *P. aeruginosa*, a correlation has been established between high ligand affinity and highly sensitive responses (Reyes‐Darias, Yang, et al., 2015b). The capacity to mediate responses to low ligand concentrations may be a feature common to the GABA‐ and l‐Pro‐responsive chemoreceptors in pseudomonads.

The PsPto‐PscC mutant showed some residual responses to both of its ligands, indicative of the existence of additional chemoreceptors that respond to these amino acids, a finding that may not surprise considering that PsPto encodes 49 chemoreceptors. Previous studies showed that GABA and l‐Pro are not preferred nutrients for PsPto and that they are only used when the preferential nutrients have been consumed (McCraw et al., 2016). The described chemoattraction to these compounds, regardless of their nutritional value, may indicate that PsPto uses the perception of these amino acids as a cue to localize the apoplast and initiate infection. Similarly, the use of nonpreferred amino acids as signals to locate nutrients and start the interaction with the plant has been previously proposed for *Bacillus subtilis* (Yang et al., 2015).

The PsPto genome encodes three GABA transaminases and three succinate‐semialdehyde dehydrogenases (Park et al., 2010). However, PsPto is unable to synthetize GABA directly from glutamate due to the lack of a GadB enzyme (McCraw et al., 2016). Therefore, it is tempting to speculate that this bacterium obtains GABA from the plant, particularly from the apoplast, where it is more abundant. The unusually high number of GABA catabolic enzymes in the PsPto genome may be related to a particular importance of GABA metabolism during the interaction with the plant host.

In a previous transcriptomic analysis carried out in *P. syringae* pv. *syringae* B728a, it was observed that the expression of *gabP* and GABA catabolic genes was up‐regulated in planta and that their expression was increased in cells growing in the apoplast as compared to epiphytic populations (Yu et al., 2013). We show here that the addition of GABA to PsPto growing in *hrp*‐inducing minimal medium, increased *gabP* expression. GabP represents the main mechanism of GABA uptake in PsPto (McCraw et al., 2016). We also observed up‐regulation of the encoding succinate‐semialdehyde dehydrogenase (*gabD*) and transaminases (*gabT1* and *gabT2*), but not of *gabT3*. It has been suggested that the *gabT3* gene, found in a different location of the genome, may not be a GABA transaminase (Park et al., 2010). Interestingly, we observed that in the presence of GABA, the expression of *gabD*, *gabT1*, and *gabT2*, but not that of *gabP*, was down‐regulated in the PsPto‐*pscC* mutant strain, which suggests that the perception of GABA through this chemoreceptor regulates the expression of GABA catabolism genes.

The expression of T3SS genes, the main virulence factor in PsPto, is induced in the presence of plant‐derived signals (Anderson et al., 2014; Tang et al., 2006). A recent work has reported that the two‐component system AauS–AauR in PsPto directly connects the perception of host‐derived amino acids (aspartate and glutamate) to the up‐regulation of T3SS‐encoding genes (Yan et al., 2020). Interestingly, the presence of increased levels of GABA down‐regulates the expression of T3SS‐related genes in PsPto (Park et al., 2010). Accordingly, we found that the expression of *avrPto* and *hrpL* genes was down‐regulated in the WT strain in the presence of 1 mM GABA and that this regulation was not mediated by PsPto‐PscC. In PsPto, the repression of T3SS‐related genes is mediated by GABA uptake through the GabP permease (McCraw et al., 2016) and the expression of *gabP* was similar in the WT and the PsPto‐*pscC* mutant strain. Interestingly, the expression of the *B. subtilis* GABA catabolic genes *gabT* and *gabD* is under the control of the regulatory protein GabR; however, the expression of *gabP* is not regulated by this protein (Belitsky & Sonenshein, 2002).

The genome analysis of PsPto indicates that it has four chemosensory pathways (Gumerov et al., 2019) and currently available data indicate that only one is involved in chemotaxis (Cerna‐Vargas et al., 2019; Clarke et al., 2016). In *P. aeruginosa*, the four chemosensory pathways appear to be insulated signalling routes (Matilla, Martin‐Mora, et al., 2021; Ortega, Fleetwood, et al., 2017). However, results have been reported that suggest a cross‐talk between different chemosensory pathways in other species (Cerna‐Vargas et al., 2019; Huang et al., 2019). Importantly, our previous analysis of the PsPto‐PscA chemoreceptor strongly suggests the existence of interpathway communication (Cerna‐Vargas et al., 2019). We demonstrated that deletion of the PsPto‐*pscA* gene not only reduced chemotaxis to its cognate ligands, but also increased cyclic‐di‐GMP levels, which in turn modulated biofilm formation and swarming motility. We hypothesize that a similar cross‐talk may account for the observation that the mutation of the PsPto‐PscC chemoreceptor modulates the expression of genes involved in GABA catabolism.

We propose that PsPto‐PscC has a role as GABA chemoreceptor in the early stages of plant interaction, namely the epiphytic phase, to locate entry sites and navigate towards them, ensuring apoplast invasion. Consistently, we show here that the reduced chemotactic response towards GABA and l‐Pro observed in the mutant strain caused an impaired leaf entry and reduced virulence in tomato plants. We did not observe differences either in entry or in virulence between the WT and the mutant strain in *A. thaliana* plants. PsPto infected *A. thaliana* WT and *gad1/2* mutant plants, which produce lower GABA levels, in a similar manner. This is consistent with the proposition that PsPto virulence in *Arabidopsis* is not influenced by GABA (Xian et al., 2020), as it is the case in tomato plants. Under nonstress conditions GABA levels are lower in *Arabidopsis* than in tomato (Deng et al., 2020; Rico & Preston, 2008). Although GABA levels can suffer a threefold increase during biotic stress in *Arabidopsis* (Ward et al., 2010), the concentration is still well below that found in the tomato apoplast, which is around 500 μM (Rico & Preston, 2008) under nonstress conditions and may increase to 2.5 mM under biotic stress (Solomon & Oliver, 2001). These data suggest that the signals perceived by PsPto during infection of tomato and *Arabidopsis* are different.

Our data highlight the key role of GABA and l‐Pro as plant‐released chemoeffectors perceived by PsPto to drive entry into the tomato host plant. These results are in line with the suggested pivotal role of amino acid perception during bacterial plant infection. The differential role of this perception in *Arabidopsis* plants suggests that the specific profile of pathogen chemoperception functions is under evolutionary pressure of the presence of host‐specific signals.

## 4 EXPERIMENTAL PROCEDURES

### 4.1 Bacterial strains, culture media, and growth conditions

Bacteria and plasmids used in this study are listed in Table S1. PsPto derivatives were grown at 28°C in King's B (KB) medium (King et al., 1954). *Escherichia coli* derivatives were grown at 37°C in lysogeny broth (LB) (Bertani, 1951). When appropriate, antibiotics were added to the medium at the following final concentrations: rifampicin 25 μg/ml, streptomycin 50 μg/ml, kanamycin 50 μg/ml.

### 4.2 Construction of the expression plasmid for PsPto‐PscC‐LBD

The limits of the LBD domain of PsPto‐PscC (PSPTO_2448) were defined as the protein fragment between the two transmembrane regions as determined by the TMHMM server v. 2.0 (Krogh et al., 2001) and the DAS transmembrane region prediction algorithm (Cserzo et al., 1997).

A pET28b(+) derivative containing the DNA sequence of the PsPto‐PscC‐LBD, p2448‐LBD, was purchased from Biocat. *E. coli* BL21(AI) (Invitrogen) was transformed with this plasmid for protein overexpression.

### 4.3 Overexpression and purification of PsPto‐PscC‐LBD

*E. coli* BL21(AI) containing p2448‐LBD was grown in LB medium supplemented with kanamycin at 28°C. Once the culture reached an OD_600_ of 0.5, protein overexpression was induced by the addition of 0.05 mM isopropyl‐β‐d‐1‐thiogalactopyranoside and l‐arabinose 0.2% (wt/vol). Growth was then continued overnight at 16°C prior to cell harvest by centrifugation at 6000 × *g* for 30 min at 4°C. Cell pellets were resuspended in buffer A (30 mM Tris.HCl, 300 mM NaCl, 10 mM imidazole, 10% [vol/vol] glycerol, pH 8.0), disrupted by sonication, and centrifuged at 13,000 × *g* for 1 h. The supernatant was then loaded onto a 5‐ml HisTrap column (Amersham Biosciences) previously equilibrated with 5 column volumes of buffer A, which was subsequently washed with buffer A containing 10 mM imidazole and eluted with a linear gradient of 10 to 500 mM imidazole in buffer A. Protein‐containing fractions were pooled and dialysed into buffer B (50 mM HEPES, 300 mM NaCl, 10% [vol/vol] glycerol, pH 8.0) for immediate analysis.

### 4.4 Thermal shift assay‐based high‐throughput ligand screening

Thermal shift assays were performed on a Bio‐Rad MyIQ2 real‐time PCR instrument using 96‐well plates. For ligand screening, the compound array PM3B (nitrogen sources) from Biolog (https://www.biolog.com/wp‐content/uploads/2020/04/00A‐042‐Rev‐C‐Phenotype‐MicroArrays‐1‐10‐Plate‐Maps.pdf) was used. Assay mixtures (25 μl) contained 15 μM protein dialysed into buffer B, 5× SYPRO Orange (Life Technologies), and ligands at final concentrations of 1 and 5 mM (1 to 2 mM when using Biolog plates). In the ligand‐free control well, the corresponding amount of buffer was added. Samples were heated from 23°C to 85°C at a scan rate of 1°C/min. The protein unfolding curves were obtained by monitoring the changes in SYPRO Orange fluorescence. *T*_*m*_ values were determined using the first derivatives of the raw fluorescence data.

### 4.5 Isothermal titration calorimetry binding studies

Experiments were conducted on a VP‐ITC microcalorimeter (MicroCal) at 25°C. PsPto‐PscC‐LBD was dialysed overnight against buffer B, adjusted to a concentration of 13 μM, and placed into the sample cell of the instrument. The protein was titrated by the injection of 4.2–8.6‐μl aliquots of 500 μM ligand solutions that were prepared in buffer B immediately before use. The mean enthalpies measured from the injection of ligands into buffer were subtracted from raw titration data prior to data analysis using the “one‐binding‐site” model of the MicroCal version of ORIGIN.

### 4.6 Construction of PsPto‐*pscC* mutant

To generate the PsPto‐*pscC* mutant, an internal 502‐bp fragment of *PSPTO_2448* was amplified by PCR from the PsPto genome using the corresponding primers (Table S2). PCR products were cloned into pKOSac101 (Cerna‐Vargas et al., 2019) and the resulting plasmid was introduced in *E. coli* CC118λ*pir*, purified, and transferred into PsPto by electroporation. Plasmid integration was confirmed using PCR. For complementation assays, the chemoreceptor‐encoding gene and its promoter region were amplified by PCR using the primers listed in Table S2 and the PCR product was cloned into the broad‐host‐range plasmid pBBR1MCS‐2 (Kovach et al., 1995). The resulting plasmid was verified by DNA sequencing and transferred into the PsPto‐*pscC* mutant by electroporation.

### 4.7 Quantitative capillary chemotaxis assays

Bacterial cells were cultured overnight at 28°C in KB medium and diluted to an OD_600_ of 0.05. At the early stationary phase of growth (OD_600_ of 0.25), cultures were centrifuged at 1750 × *g* for 5 min, and the resulting pellet was washed twice with 10 mM HEPES, pH 7.0. Cells were then resuspended in the same buffer and adjusted to an OD_600_ of 0.25. Subsequently, 230 μl of the cell suspension was placed into each well of a 96‐well plate. One‐microlitre capillaries were filled with the chemoeffector and immersed into the bacterial suspension for 30 min. Capillaries were removed from the bacterial suspension and rinsed with sterile water, and the content was expelled into 1 ml of NB medium (1 g/L yeast extract, 2 g/L beef extract, 5 g/L NaCl, and 5 g/L Bacto peptone). Serial dilutions were plated onto NB medium supplemented with the appropriate antibiotics, and the number of cfu was determined. In all cases, data were corrected by subtracting the number of cells that swam into buffer‐containing capillaries.

### 4.8 RNA extraction

Bacterial cells were grown at 28°C in *hrp*‐inducing medium (Huynh et al., 1989) with shaking at 150 rpm to an OD_600_ of 0.5. When necessary, GABA at a final concentration of 1 mM was added and cultures were incubated at 28°C for 2 h. Cells were then collected by centrifugation at 5000 × *g* for 5 min, the supernatant was discarded, and the pellets were stored at −80°C. Total RNA was extracted using TRI reagent solution (ThermoFisher Scientific) following the manufacturer's instructions. The purification was accomplished using the High Pure RNA Isolation Kit (Roche) following the manufacturer's recommendations. RNA samples were quantified using a NanoDrop 1000 spectrophotometer (NanoDrop Technologies, Inc.).

### 4.9 Reverse transcription‐quantitative PCR

Total RNA was isolated as described above and converted to cDNA using a High‐Capacity cDNA Reverse Transcription Kit (Applied Biosystems). Primers were designed to amplify fragments of approximately 100 bp (Table S2) and the *rpoD* gene was used as an internal control (Sawada et al., 1999). Reverse transcription‐quantitative PCR (RT‐qPCR) amplifications were carried out on an ABI PRISM 7300 RT PCR System (Applied Biosystems) using SYBR Green PCR Master Mix (Applied Biosystems). Thermal cycling conditions were as follows: one cycle at 95°C for 10 min; 50 cycles at 95°C for 15 s and 60°C for 1 min; and a final cycle at 95°C for 15 s, 60°C for 1 min, 95°C for 15 s, and 60°C for 15 s. The relative gene expression ratio was calculated using the comparative critical threshold (ΔΔ*C*_t_) method (Pfaffl, 2001; Rotenberg et al., 2006).

### 4.10 Plant assays

*P. syringae* pv. *tomato* strains were grown at 28°C for 24 h on KB agar plates under dark conditions.

For virulence assays in tomato, cells were resuspended in 10 mM MgCl_2_ and diluted to 1 × 10^8^ cfu/ml. Three‐week‐old tomato plants (*Solanum lycopersicum* ‘Moneymaker’) were spray‐inoculated with the bacterial suspension. Silwet L‐77 was added to the bacterial suspensions at a final concentration of 0.02% (vol/vol). Plants were infected at subjective dawn, which was at the end of the night cycle with immediate transfer to light. Plants were incubated in a growth chamber at 25°C at 60% relative humidity with a daily light period of 12 h. Six days after inoculation, the leaf symptoms were recorded, and bacterial populations were measured by sampling five 1‐cm‐diameter leaf discs per plant. Three independent biological replicates were performed. In each replicate three plants were used, and five independent leaves were sampled from each plant. The infected leaf discs were washed twice with 10 mM MgCl_2_ prior to homogenization to eliminate the bacteria from the leaf surface. Plant material was homogenized in 10 mM MgCl_2_ and drop‐plated onto KB agar supplemented with the appropriate antibiotics. The average number of bacteria per cm^2^, isolated from five infected tomato leaves, was determined based on log‐transformed data.

For the infiltration assays, leaves were syringe‐infiltrated with a suspension containing 3 × 10^4^ cfu/ml, and three leaves were mock‐infiltrated with 10 mM MgCl_2_. Bacterial populations were determined at 3 dpi following the same protocol described above for the spray‐inoculation assay.

Tomato entry assays were performed as described above for the spray‐inoculation and the syringe‐infiltration assays, using a bacterial suspension containing 1 × 10^8^ cfu/ml. Bacterial populations were determined at 2 h postinoculation. For entry assays, a surface sterilization procedure (Romero et al., 2019) was carried out prior to plant material homogenization. Briefly, leaves were surface‐sterilized with a 5% (vol/vol) solution of commercial bleach and 0.01% (vol/vol) of Tween 20 for 10 min and finally rinsed three times with sterile distilled water. The final wash was plated on KB plates to confirm the absence of bacteria.

For virulence assays in *A. thaliana*, cells were resuspended in 10 mM MgCl_2_ and diluted to 3 × 10^8^ cfu/ml. Four‐week‐old *A. thaliana* Col‐0 or *gad1/2* double mutant (Scholz et al., 2015) plants were spray‐inoculated with the bacterial suspension. The symptoms were recorded at 6 dpi. Bacterial populations on the leaves were determined by quantifying the average number of cfu isolated from three leaves. Plant material was processed as described for tomato plants.

### 4.11 Statistical analysis

Variables that were heteroscedastic and that did not follow a normal distribution were compared using generalized linear models. The remaining variables were analysed using general linear models. All analyses were performed using the statistical software package SPSS 25.0 (SPSS Inc.) and Centurion 18 (Statgraphics Technologies, Inc.).

## Supporting information


**FIGURE S1** Differential scanning fluorimetry‐based ligand screening of PsPto‐PscC‐LBD. Shown are the changes in the midpoint of the protein unfolding transition (*T*_*m*_) for the compounds present in the Biolog PM3B phenotype microarray of nitrogen sources with respect to the *T*_*m*_ of the ligand‐free protein. The dashed line indicates the threshold of 2°C for significant hits

**FIGURE S2** Quantitative capillary chemotaxis assays of *Pseudomonas syringae* pv. *tomato* (WT) and the PsPto‐*pscC* mutant towards l‐Asp, l‐Glu, and l‐Asn at 1 mM. The data have been corrected with the number of cells that swam into buffer‐containing capillaries. Shown are means and standard errors from three independent experiments

**FIGURE S3** Quantitative capillary chemotaxis assays of *Pseudomonas syringae* pv. *tomato* (WT), the PsPto‐*pscC* mutant, and the complemented mutant (PsPto‐*pscC* Comp) towards GABA and l‐Pro at 1 and 5 mM. The data have been corrected with the number of cells that swam into buffer‐containing capillaries. Shown are means and standard errors from three independent experiments conducted in triplicate. Values that are significantly different are indicated by asterisks (*p < 0.05; **p < 0.01; ***p < 0.005; ****p < 0.001)

**FIGURE S4** Bacterial populations of *Pseudomonas syringae* pv. *tomato* (WT), the PsPto‐*pscC* mutant, and the complemented mutant (PsPto‐*pscC* Comp) at 6 days postinoculation after spray‐inoculation (10^8^ cfu/ml) in tomato plants. Shown are means and standard errors from two independent biological replicates. Values that are significantly different are indicated by asterisks (*p < 0.05)

**TABLE S1** Bacteria and plasmids used

**TABLE S2** Primers used in this study

## Data Availability

The data that support the findings of this study are available from the corresponding author upon reasonable request.
