# Supplementary figures and images for: Pseudomonas syringae pv. tomato infection of tomato plants is mediated by GABA and l‐Pro chemoperception
Source: Mol Plant Pathol. 2022 Jun 10;23(10):1433–45. doi: 10.1111/mpp.13238 (PMC9452764; doi:10.1111/mpp.13238)

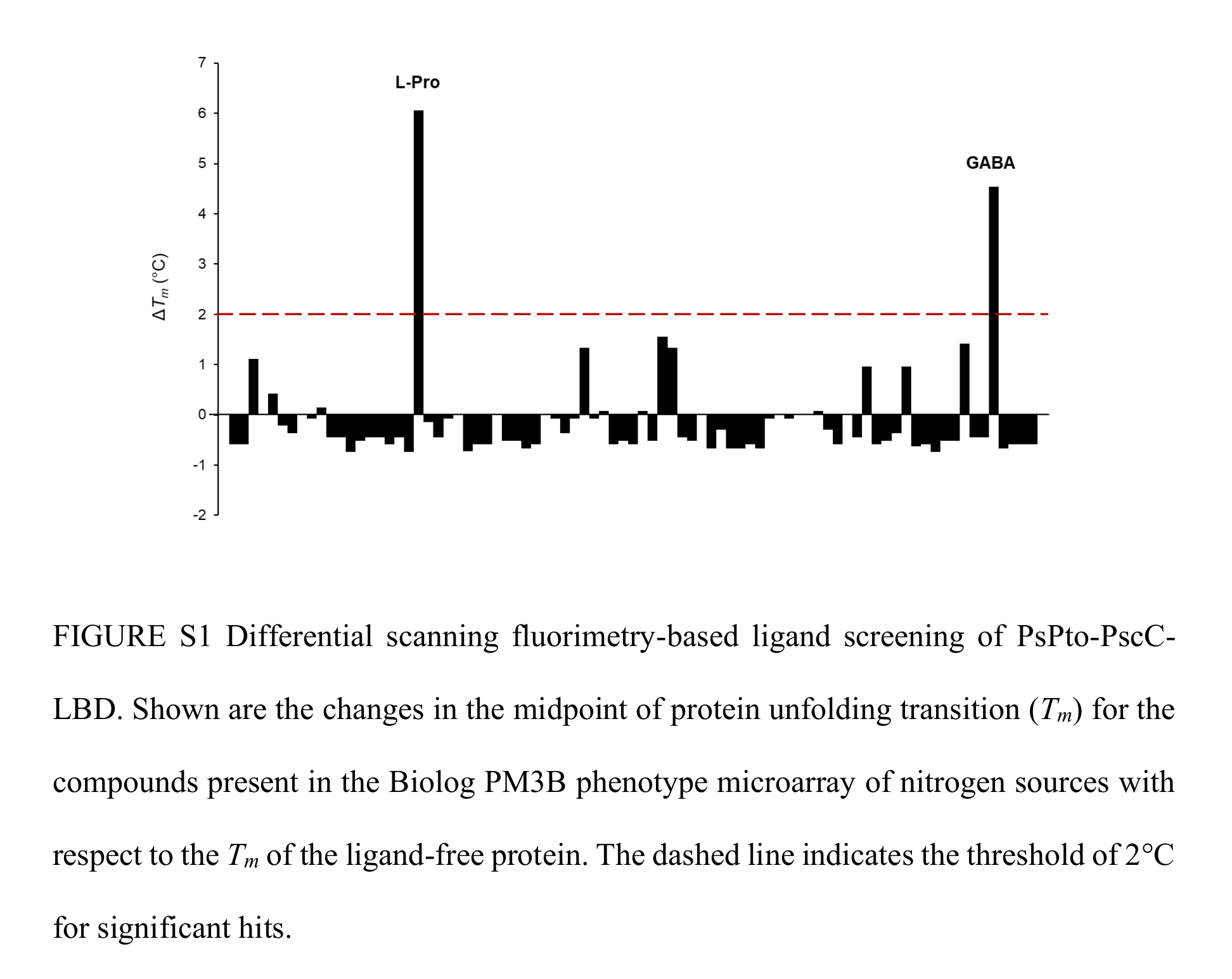

Supplement: Supplementary file 1 — FIGURE S1 Differential scanning fluorimetry‐based ligand screening of PsPto‐PscC‐LBD. Shown are the changes in the midpoint of the protein unfolding transition (T m ) for the compounds present in the Biolog PM3B phenotype microarray of nitrogen sources with respect to the T m of the ligand‐free protein. The dashed line indicates the threshold of 2°C for significant hits [file MPP-23-1433-s002.tiff]

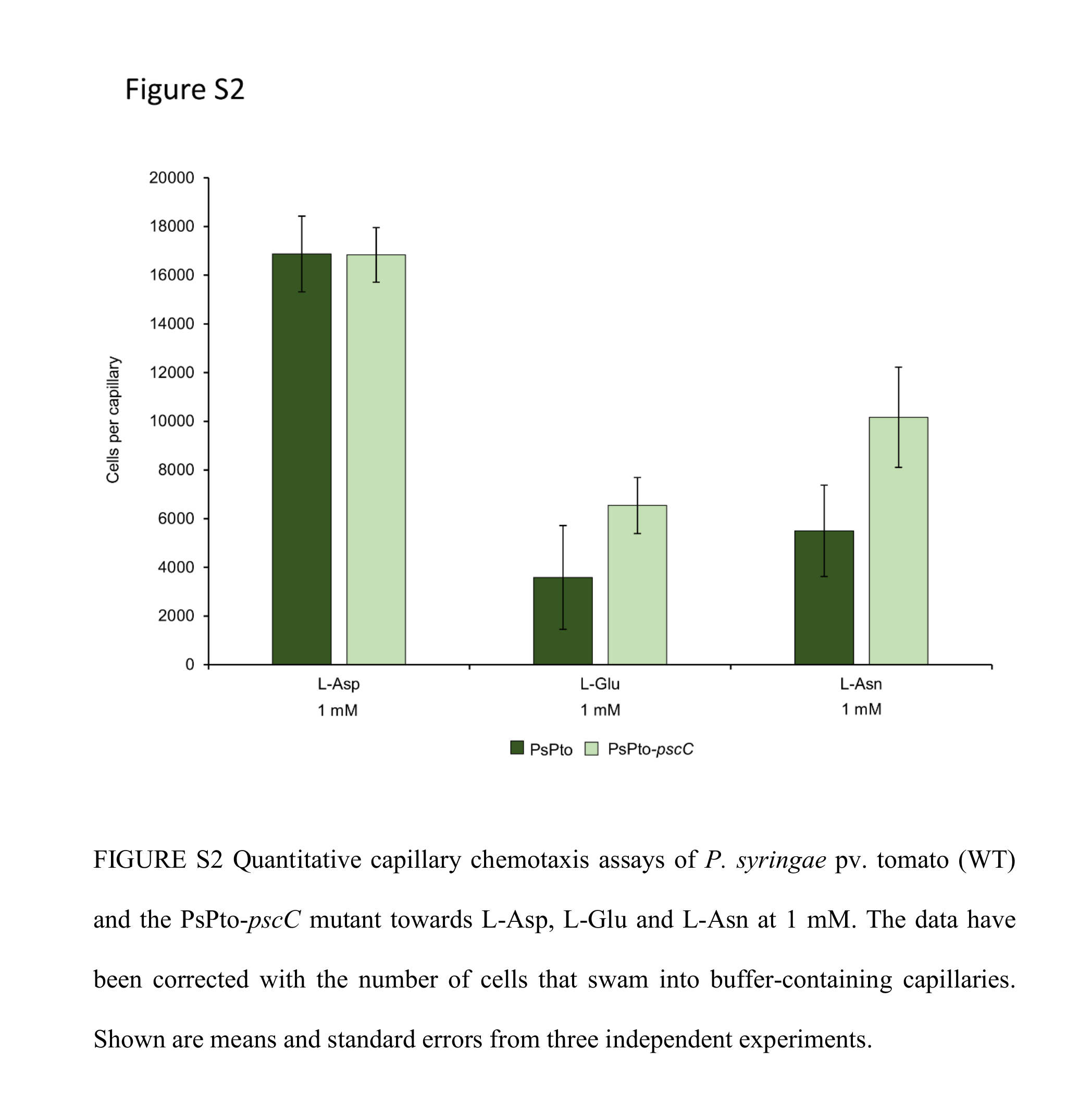

Supplement: Supplementary file 2 — FIGURE S2 Quantitative capillary chemotaxis assays of Pseudomonas syringae pv. tomato (WT) and the PsPto‐pscC mutant towards l‐Asp, l‐Glu, and l‐Asn at 1 mM. The data have been corrected with the number of cells that swam into buffer‐containing capillaries. Shown are means and standard errors from three independent experiments [file MPP-23-1433-s004.tiff]

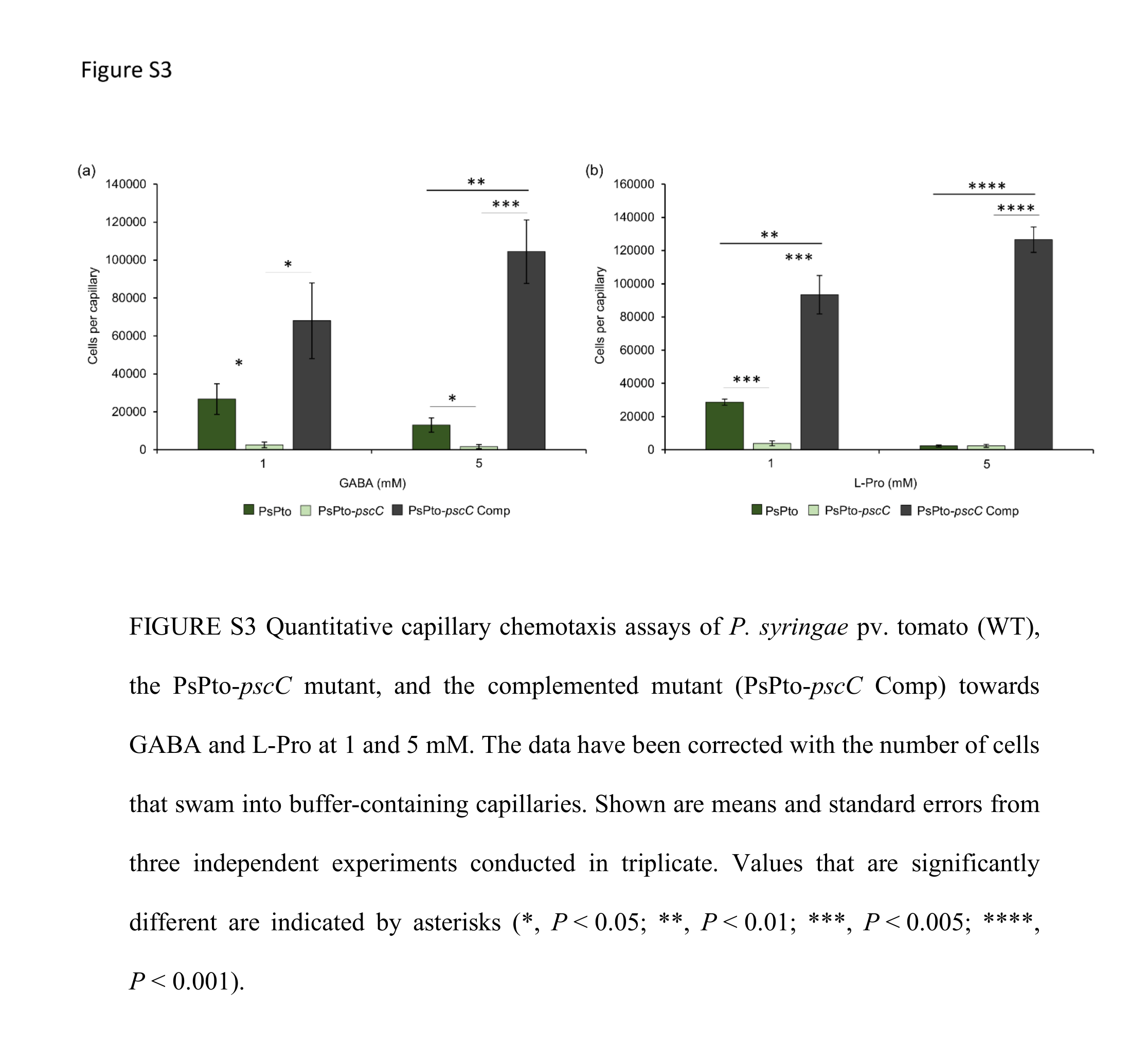

Supplement: Supplementary file 3 — FIGURE S3 Quantitative capillary chemotaxis assays of Pseudomonas syringae pv. tomato (WT), the PsPto‐pscC mutant, and the complemented mutant (PsPto‐pscC Comp) towards GABA and l‐Pro at 1 and 5 mM. The data have been corrected with the number of cells that swam into buffer‐containing capillaries. Shown are means and standard errors from three independent experiments conducted in triplicate. Values that are significantly different are indicated by asterisks (*p < 0.05; **p < 0.01; ***p < 0.005; ****p < 0.001) [file MPP-23-1433-s003.tiff]

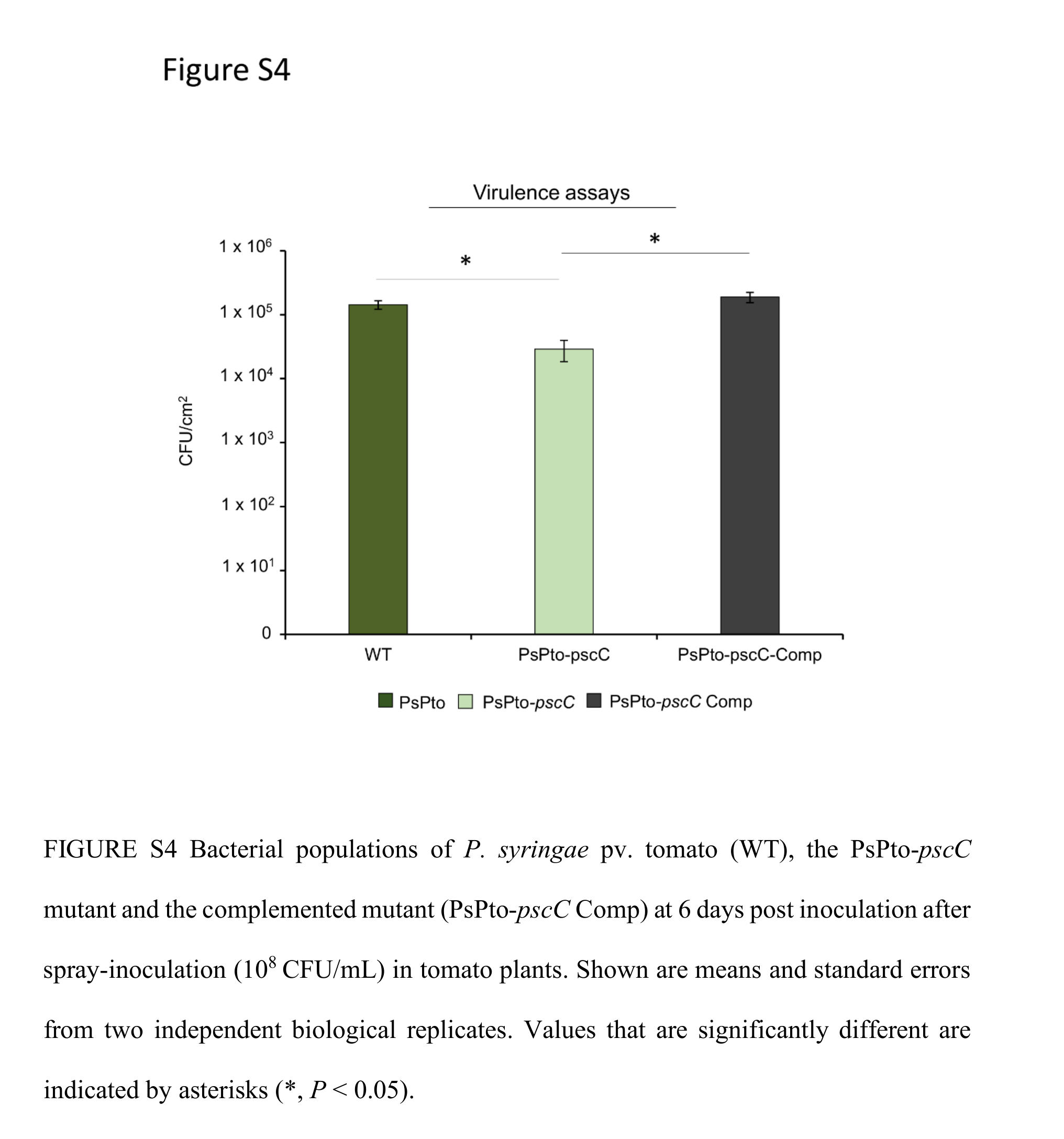

Supplement: Supplementary file 4 — FIGURE S4 Bacterial populations of Pseudomonas syringae pv. tomato (WT), the PsPto‐pscC mutant, and the complemented mutant (PsPto‐pscC Comp) at 6 days postinoculation after spray‐inoculation (108 cfu/ml) in tomato plants. Shown are means and standard errors from two independent biological replicates. Values that are significantly different are indicated by asterisks (*p < 0.05) [file MPP-23-1433-s001.tiff]
