# Supplementary material for: Pseudomonas syringae pv. tomato infection of tomato plants is mediated by GABA and l‐Pro chemoperception
Source: Mol Plant Pathol. 2022 Jun 10;23(10):1433–45. doi: 10.1111/mpp.13238 (PMC9452764; doi:10.1111/mpp.13238)
Supplement: Supplementary file 5 — TABLE S1 Bacteria and plasmids used [file MPP-23-1433-s006.pdf]

Table S1. Bacteria and plasmids used.

Strains and plasmids	Relevant characteristics[a]	Reference or source
E. coli		
CC118λ*pir*	Sp^r, Δ(*ara-leu*) *araD* Δ*lacX*74 *galE galK phoA*20 *thi-1 rpsE rpoB argE recA*1 lysogenized with λ*pir* phage	(Herrero et al., 1990)
DH5α	*supE*44 *lacU*169 (*Ø80lacZΔM15*) *hsdR*17 (r_k-m_k-) *recA*1 *endA*1 *gyrA*96 *thi*-1 *relA*1	(Hanahan, 1983)
BL21 (AI)	*F^- ompT gal dcm lon hsdS$_B$($r_B^-m_B^-$) λ(DE3 [lacI lacUV5-T7p07 ind1 sam7 nin5]) [malB$^+$]$_{K\text{-}12}$(λ^S)*	Invitrogen, CA, USA
***P. syringae* pv. tomato DC3000**		
WT	Rif^r, wild type	(Cuppels, 1986)
PsPto-*pscC*	Sm^r, *PSPTO_2448*::pKOSac101	This study
PsPto-*pscC* Comp	Sm^r; Km^r PsPto-*pscC* complemented with pBBR1MCS-2-2448	This study
**Plasmids**		
pET28b(+)	Km^r, His-tagged protein expression vector	Novogene
p2448-LBD	Km^r, N-terminal His-tagged PSPTO_2448-LBD expression vector	This study
pKOSac101	Sm^r, *oriR6K mob* pKNG101 harboring an 144-bp internal deletion of *sacB*	(Cerna-Vargas et al., 2019)
pKOSac101-2448	Sm^r, pKOSac101 with a 502-bp PCR fragment of *PSPTO_2448* cloned at XmaI sites	This study
pBBR1MCS-2	Km^r, broad-host-range plasmid *oriT*RK2 *mob*RK2	(Kovach et al., 1995)
pBBR1MCS-2-2448	Km^r; a 2.2-kb PCR fragment containing the *PSPTO_2448* gene and its promoter region	This study

[a] Km, kanamycin; Rif, rifampicin; Sm, streptomycin.

Cerna-Vargas, J. P., Santamaría-Hernando, S., Matilla, M. A., Rodríguez-Herva, J. J., Daddaoua, A., Rodríguez-Palenzuela, P., Krell, T., and López-Solanilla, E. (2019). Chemoperception of specific amino acids controls phytopathogenicity in *Pseudomonas syringae* pv. tomato. *mBio* **10**, e01868-19.

Cuppels, D. A. (1986). Generation and Characterization of Tn*5* Insertion Mutations in *Pseudomonas syringae* pv. *tomato*. *Appl Environ Microbiol* **51**, 323-327.

Hanahan, D. (1983). Studies on transformation of *Escherichia coli* with plasmids. *J Mol Biol* **166**, 557-580.

Herrero, M., de Lorenzo, V., and Timmis, K. N. (1990). Transposon vectors containing non-antibiotic resistance selection markers for cloning and stable chromosomal insertion of foreign genes in gram-negative bacteria. *J Bacteriol* **172**, 6557-67.

Kovach, M. E., Elzer, P. H., Hill, D. S., Robertson, G. T., Farris, M. A., Roop, R. M., 2nd, and Peterson, K. M. (1995). Four new derivatives of the broad-host-range cloning vector pBBR1MCS, carrying different antibiotic-resistance cassettes. *Gene* **166**, 175-6.
