# Supplementary material for: Pseudomonas syringae pv. tomato infection of tomato plants is mediated by GABA and l‐Pro chemoperception
Source: Mol Plant Pathol. 2022 Jun 10;23(10):1433–45. doi: 10.1111/mpp.13238 (PMC9452764; doi:10.1111/mpp.13238)
Supplement: Supplementary file 6 — TABLE S2 Primers used in this study [file MPP-23-1433-s005.pdf]

Table S2. Primers used in this study.

Primer	Sequence (5'-3')	Purpouse
2448XmaFw	AACCCGGGCCTACGTTTCTTACGGTC	Construction of PsPto-*pscC* mutant
2448XmaRev	TTCCCGGGTTGATCAGGCCGATAAAGT	Construction of PsPto-*pscC* mutant
2448CompFw	AAAAGAATTCGCGTGCGGCACGTCC	Construction of plasmid for PsPto-*pscC* mutant complementation
2448CompRev	TTTTGAATTCCTACGCTCCAGCAGGAATG	Construction of plasmid for PsPto-*pscC* mutant complementation
rpoDFw	CGGCATCGACATGAATACCG	RT-qPCR
rpoDRev	GCGCAGTGCCTTGGCTTC	RT-qPCR
gabT1Fw	ACTCCTCATCTGCTCCGCCAG	RT-qPCR
gabT1Rev	CCGTCGACGTCCCACAGTTCA	RT-qPCR
gabT2Fw	TCCCTGATGCAACGCCGC	RT-qPCR
gabT2Rev	CGATGAATTCACGACCTTCCACGTC	RT-qPCR
gabT3Fw	TCCACAGCATGACCTTGTCCCAC	RT-qPCR
gabT3Rev	GCCCCAGATTGAGCACGCC	RT-qPCR
gabD1Fw	AGACCCCAGCCTGCTGGTG	RT-qPCR
gabD1Rev	CGATCACTTCGCCGGTCGC	RT-qPCR
gabPFw	TAGCGTGAGTGGCGTCAGTCA	RT-qPCR
gabPRev	ACGATCTCGGTGCCCATGAACG	RT-qPCR
hrpLFw	CATCTTCGTCCGCCGGTATTC	RT-qPCR
hrpLRev	CGCACTGGAGAATGTCATCCAC	RT-qPCR
avrPtoFw	GACATGCAGCATAGGTACATGAC	RT-qPCR
avrPtoRev	GCTTCGCGCATGTCACTC	RT-qPCR
